# Are we there yet? An update on transitional care in rheumatology

**DOI:** 10.1186/s13075-017-1502-y

**Published:** 2018-01-11

**Authors:** Janet E. McDonagh, Albert Farre

**Affiliations:** 1 Centre for Musculoskeletal Research, Division of Musculoskeletal and Dermatological Sciences, School of Biological Sciences, Faculty of Biology, Medicine and Health, Manchester, UK; 2 NIHR Manchester Musculoskeletal Biomedical Research Centre, University of Manchester, Manchester, UK; 3 Manchester University Hospitals NHS Foundation Trust, Manchester Academic Health Science Centre, Manchester, UK; 4 Institute of Applied Health Research, University of Birmingham, Birmingham, UK; 5 Research and Development, Birmingham Children’s Hospital, Birmingham Women’s and Children’s Hospital NHS Foundation Trust, Birmingham, UK

**Keywords:** Transitional care, Rheumatic disease, Adolescents, Young adults, Transition programme

## Abstract

Significant progress has been made in the understanding of transitional care in rheumatology over the last few decades, yet universal implementation has not been realised and unmet needs continue to be reported. Possible explanations for this include lack of evidence as to which model is most effective; lack of attention to the multiple dimensions, stakeholders and systems involved in health transitions; and lack of consideration of the developmental appropriateness of transition interventions and the services/organisations/systems where such interventions are delivered.

Successful transition has major implications to both the young people with juvenile-onset rheumatic disease and their families. Future research in this area will need to reflect both the multidimensional (biopsychosocial) and the multisystemic (multiple systems and stakeholders across personal/social/family support networks and health/social care/education systems). Only then will we be able to determine which aspects of transition readiness and service components influence which dimension. It is therefore imperative we continue to research and develop this area, involving both paediatric and adult rheumatology clinicians and researchers, remembering to look beyond both the condition and our discipline. Neither should we forget to tap into the exciting potential associated with digital technology to ensure further advances in transitional care are brought about in and beyond rheumatology.

## Background

It is now 25 years since the first transitional care programme in rheumatology was reported and a decade since the first objective evaluation of such a programme [1]. Since then other programmes have been described [2] and international guidelines developed [3], all learning from similar progress elsewhere [4, 5]. In spite of this, universal implementation has not been realised and unmet needs continue to be reported [6]. The aims of this paper are to explore why this should be, consider the current shortcomings of the existing evidence, and propose an itinerary for the next leg of the transitional care research journey.

## Main text

The rationale and aims of transitional care with juvenile-onset rheumatic disease (jRMD) are well established [1–3] and the evidence of need for transitional care in rheumatology is no longer disputed [3]. Why then are unmet needs in this area still reported [6]?

Various transition models and approaches to its evaluation have been reported in the literature [1, 2, 4]. However, it is not yet known which model is the most effective and it remains unclear how to measure the efficacy and outcomes of such a multifaceted and protracted intervention, which extends from early adolescence through to the third decade. A Delphi study involving a range of professionals identified 10 outcomes, the majority of which were health service related except for two patient-reported outcomes, ‘optimal quality of life’ and ‘a social network’, and none included vocational or psychological outcomes [5]. Similarly, randomised controlled trials to date have primarily employed health service-related measures [4]. Thus, existing outcome measures still fail to reflect all aspects of transitional care: biological (disease), psychological, social and educational/vocational. Furthermore, the lack of accepted transition outcome measures further hampers transition research [5].

Likewise, various transition readiness measures and checklists have been reported in the literature [7], and although their use is advocated in current guidance [3] they are not used universally in practice [6]. Two key challenges are: due to the interrelated nature of adolescent development, measures are likely to reflect transition readiness trajectories over the broader life stage, rather than selectively reflecting healthcare transition readiness; and it is largely unknown as to when such readiness skills should be developing. Furthermore, some measures fail to encompass all aspects of adolescent development relevant to healthcare transitions. For example, not all measures used in European rheumatology centres consider educational and vocational development [6], which is concerning in view of the vocational morbidity of jRMD in this age group [8]. Also, currently available tools are based on self-reporting, which may not evaluate all aspects of mastery of ‘transition’ skills. Finally, consideration of the setting (whether health, home and/or school) in which young people practise these skills is also important. A young person may be ‘transition-ready’ but if a particular setting does not support/promote positive youth development, the development of such skills may be delayed due to lack of opportunity.

A frequently reported barrier to implementation includes lack of resources. A key resource is the availability of trained staff. There is a significant association between youth perceptions of autonomy support from their rheumatologist and healthcare transition discussions [9]. The use of the HEEADSSS tool has been advocated by several authors, and is now referenced (alongside appropriate training in adolescent health) in the EULAR recommendations [3, 6, 9]. However, potential efficacy of current training should be considered in view of reported discrepancies between professionals and young people as to which topics are actually discussed in clinics [10]. Such issues should be taken into consideration going forward.

In view of the generic nature of the developmental needs of young people with long-term health conditions, one could argue that transitional care programmes can be generic rather than disease specific, with the resulting potential efficiencies of scale in terms of resources and funding. Likewise, research should consider a generic approach whether within disciplines or across disciplines. The latter is particularly important so as to learn from other disciplines and thereby not slow down progress in this field. Alongside this, further improvement can be facilitated by digital technology, which is still perceived as underused in transitional care [6, 11].

A key area less studied to date is the health system/regulatory framework where such care is being provided. Young people experience health transitions across a range of settings, not just rheumatology clinics. Flexibility, funding and cross-sectoral collaboration (including communication and coordination) at the system level have been identified as areas in need of further improvement [12]. The need to secure funding for provision of transitional care is a welcome inclusion in the EULAR recommendations [3].

Another less researched area is consideration of how developmentally appropriate a particular programme/service is, including the institution it is delivered within. Too often, health transition is considered merely as the negotiation of the structural boundaries between child and adult services rather than a holistic approach to the developmental needs of young people as they grow up and negotiate multiple transitions of which healthcare is only one. Provision of developmentally appropriate health care [13] is a potential facilitator of health transition and is the backdrop on which health transition should always be considered [14].

## Conclusions

Young people with jRMD in the UK recently reported transition as a research priority [15] and whilst acknowledging the progress so far, there is much left to do. Future research will need to reflect both the multidimensional (biopsychosocial) and the multisystemic (multiple systems and stakeholders across personal/social/family support networks and health/social care/education systems). Only then will we be able to determine which aspects of transition readiness and service components influence which dimension. Successful transition has major implications to both the young people with jRMD and their families as well as to cohort studies and registries. It is therefore imperative we continue to research and develop this area, involving both paediatric and adult rheumatology clinicians and researchers, and not forgetting to look beyond the condition and our discipline so as not to reinvent unnecessary wheels.

## Abbreviations

jRMD: Juvenile onset rheumatic disease
EULAR: European League Against Rheumatism
HEEADSSS: Home, Education/Employment, Eating, Activities, Drugs, Sexuality, Suicidal Ideation and Safety
